# Yeast Rai1p and the RAC complex (Ssz1p/Zuo1p) modulate uORF-mediated *GCN4* translational control under stress conditions

**DOI:** 10.1261/rna.081039.126

**Published:** 2026-11

**Authors:** Kristína Jendruchová, Adriana Šubrtová, Leoš Shivaya Valášek

**Affiliations:** Laboratory of Regulation of Gene Expression, Institute of Microbiology of the Czech Academy of Sciences, Prague 142 20, Czech Republic

**Keywords:** general amino acid control, GCN4, translation reinititation, RAI1, RAC complex

## Abstract

Translation reinitiation (REI) is one of the most important gene-specific regulatory mechanisms by which eukaryotic cells influence expression of main translons, for example, during highly conserved integrated stress response (ISR). In *Saccharomyces cerevisiae*, expression of the key stress response gene, *GCN4*, is controlled by an intricate interplay among four short upstream translons (uTranslons, formerly uORFs), resulting in high or low levels of REI at *GCN4* depending on the growth conditions. Under nutrient-rich conditions, *GCN4* expression is repressed, but upon amino acid starvation, it is derepressed, despite a general translational shutdown. Capitalizing on our screening reporter system, we identified three new factors influencing efficiency of REI after translation of *GCN4* uTranslons: Rai1p (an RNA quality control and processing factor), and Ssz1p and Zuo1p (members of the ribosome associated complex [RAC]). Importantly, we showed that depletion of these factors deregulated derepression of Gcn4p synthesis under starvation. Furthermore, we found that similar to RAC, Rai1p associates with 40S subunits and actively translating ribosomes. We also explored interactomes of these proteins. Collectively, we present three previously unknown factors that coregulate stress response to amino acid starvation in the budding yeast by unique mechanisms.

## INTRODUCTION

Translation is a tightly regulated step of gene expression. It is influenced by a network of *cis*- and *trans*-acting factors that all act in concert to promote the action of the main actor in this process—the ribosome. This molecular machine is comprised of the small 40S subunit and large 60S subunit that join together to form the elongation-competent 80S ribosome. Translation initiation naturally begins the whole translational cycle, for which the translation initiation site (TIS; in the most cases AUG) of mRNA must be recognized by the anticodon of methionyl initiator tRNA (Met-tRNA_i_^Met^). Translation of most eukaryotic mRNAs depends on the so-called scanning mechanism, where the 40S subunit is first loaded with Met-tRNA_i_^Met^ in a ternary complex (eIF2-TC) with GTP-bound eukaryotic initiation factor (eIF) 2 in a reaction promoted by eIF1, eIF1A, eIF5 and the multisubunit complex eIF3. This forms the 43S preinitiation complex (PIC), which in turn is recruited to the 5′ end of the mRNA by the eIF4F complex, forming the 48S PIC. The 5′UTR of the mRNA is then inspected base-by-base for complementarity to the anticodon of Met-tRNA_i_^Met^ as the individual mRNA codons enter the 40S decoding site. Subsequent TIS recognition triggers GTP hydrolysis on eIF2, followed by the release of eIF2-GDP and eIF5 from the 40S subunit. The whole process is captured by the 60S joining step followed by GTP hydrolysis on eIF5B. Upon ejection of eIF5B-GDP coupled with eIF1A, the 80S initiation complex carries on into elongation (for review, see Hinnebusch 2017; Dever et al. 2023; Brito Querido et al. 2024). Completion of a synthesis of a given protein is marked by translation termination, followed by a two-step ribosome recycling phase to allow a new round of translation to begin.

However, in specific cases termination is followed by only a partial 60S recycling (i.e., only the first recycling step materializes), which enables another 40S-mediated initiation event to occur downstream from the just translated translon on the same mRNA molecule. (Translon [short for translated region] refers to a recently proposed alternative to the open reading frame [ORF] and coding DNA sequence [CDS], denoting any region that is genuinely decoded by the ribosome [Świrski et al. 2025]). This process is called translation reinitiation (REI) (for review, see Gunišová et al. 2018; Dever et al. 2023). REI is often utilized by the cell to regulate the rate of translation of the main translon through translation of short upstream translons (uTranslons, formerly uORFs) located in the 5′UTR region of an mRNA. Translation of uTranslons is by definition inhibitory toward translation of the main translon (Dever et al. 2023). Importance of this type of translational regulation is illustrated by the fact that around ∼49% of human (Calvo et al. 2009) and ∼13% of yeast (Lawless et al. 2009) transcripts contain uTranslons.

Mechanistically, REI can be achieved via the following ways: (i) by the partial post-termination complex (post-TC) recycling mentioned above, where the 60S subunit is removed by the action of ABCE1/Rli1p, which is followed by either spontaneous or Tma22p/DENR–Tma20p/MCTS1-mediated release of deacylated tRNA from the ribosomal P-site (Skabkin et al. 2010; Ahmed et al. 2018); or (ii) by blocking the whole recycling process, in which case REI may occur with the complete post-TC; that is, with both subunit still bound to each other, in a manner that may or may not require release of the deacylated tRNA (Gunišová et al. 2018; Hellen 2018; Dever et al. 2023).

The rate of REI is regulated by an array of *cis*- and *trans*-acting factors, including uTranslons and other specialized mRNA features, as well as various protein effectors. For instance, uTranslon length was demonstrated to inversely correlate with the REI efficiency (Luukkonen et al. 1995; Kozak 2001; Rajkowitsch et al. 2004), which was later attributed to post-initiation retention of some eIFs, like eIF3 and eIF4G, by 80S ribosomes translating shorter uTranslons (Rajkowitsch et al. 2004; Mohammad et al. 2017, 2021; Bohlen et al. 2020a; Wagner et al. 2020; Dever et al. 2023). It was demonstrated that the efficiency of this unexpected retention, and thus the REI efficiency, gradually decreases with an increasing length of translated uTranslon. On the other hand, REI efficiency increases with the increasing distance between the uTranslon stop codon and the main translon TIS (Grant et al. 1994; Gunišová et al. 2016). This is due to the increasing probability of the eIF2-TC reacquisition by the post-termination 40S over the longer distance that it has to traverse to become fully competent of recognizing the next TIS (Szamecz et al. 2008; Munzarová et al. 2011; Gunišová et al. 2016).

Hypothetically, ribosome recycling factors might also contribute to changes in REI efficiency. After the splitting of both ribosomal subunits by Rli1p/ABCE1 and removal of deacylated tRNA from the P-site by Tma22p/DENR–Tma20p/MCTS1 and/or by Tma64p/eIF2D, the latter factors also separate the 40S subunit from the mRNA (Hellen 2018; Young et al. 2018; Bohlen et al. 2020a,b). Indeed, their absence induces genome-wide accumulation of unrecycled 40S subunits at stop codons in both uTranslons and main translons, which is biased by certain penultimate codons over the others (Bohlen et al. 2020b; Young et al. 2021). It was therefore hypothesized that disassociation of only some tRNAs, that is, those corresponding to these penultimate codons, is strongly dependent on the action of the 40S-recycling factors (Ahmed et al. 2018; Bohlen et al. 2020b; Young et al. 2021). Accordingly, only the “Tma/DENR-dependent” codons showed significantly greater accumulation of unrecycled 40S subunits. It is important to note that even the least Tma-dependent codons in yeast showed at least approximately fourfold greater 40S accumulation levels in the deletion strains compared to WT (Young et al. 2021). Importantly, however, we have recently demonstrated that even though these codon preferences do apply to bulk 40S recycling, they do not translate into an equivalent change in the rate of REI in the absence of the Tma factors. In other words, this Tma-dependency does not impose the same rules for the efficiency of REI (Jendruchová et al. 2024).

Arguably, the most prominent example of translational control via regulatory uTranslons is the so-called “delayed REI.” This mechanism governs translation of Gcn4p in *Saccharomyces cerevisiae*, as the most thoroughly studied case. Gcn4p is a transcription factor that regulates the yeast's response to amino acid starvation through activation of genes falling under the general amino acid control (GAAC) pathway (for review, see Hinnebusch 1988, 2005). The *GCN4* 5′UTR contains four short uTranslons (historically called uORF1–4; therefore, we are not changing their designation), and translation of its mRNA is very sensitive to eIF2-TC levels, which vary depending on different nutrient conditions (Fig. 1A). In particular, uORF1 is efficiently translated under any conditions, and since it is generally permissive for REI, it prevents the second recycling step to allow the 40S post-TC to start traversing downstream. In nonstressed cells (with high eIF2-TC levels), most traversing ribosomes reacquire the eIF2-TC before reaching the REI-nonpermissive uORFs 3 or 4, therefore ribosomes terminating at these two uTranslons undergo full recycling and never reach the main *GCN4* translon. In contrast, under starvation conditions, the Gcn2p kinase senses low amino acid levels and phosphorylates the α subunit of eIF2. This suspends the formation of new eIF2-TCs and, as consequence, downregulates global translation rates. As a result, ribosomes traversing downstream from uORF1 are largely unable to reacquire the new eIF2-TC before reaching uORFs 3 and 4, effectively bypassing their TISs. Critically, a significant proportion of these ribosomes reacquires the eIF2-TC while on their way from uORF4 to the *GCN4* TIS, which triggers Gcn4p expression (Mueller and Hinnebusch 1986; Dever et al. 1992; Gunišová et al. 2016). A relatively very similar mechanism operates in mammals via the master regulator of mammalian integrated stress response—ATF4 (Pakos-Zebrucka et al. 2016), although certain differences involving various *cis*-acting elements have been recently reported by us and others (Sidrauski et al. 2015; Starck et al. 2016; Guan et al. 2017; Smirnova et al. 2024; Andreev et al. 2025; Chen et al. 2025).

**FIGURE 1.**
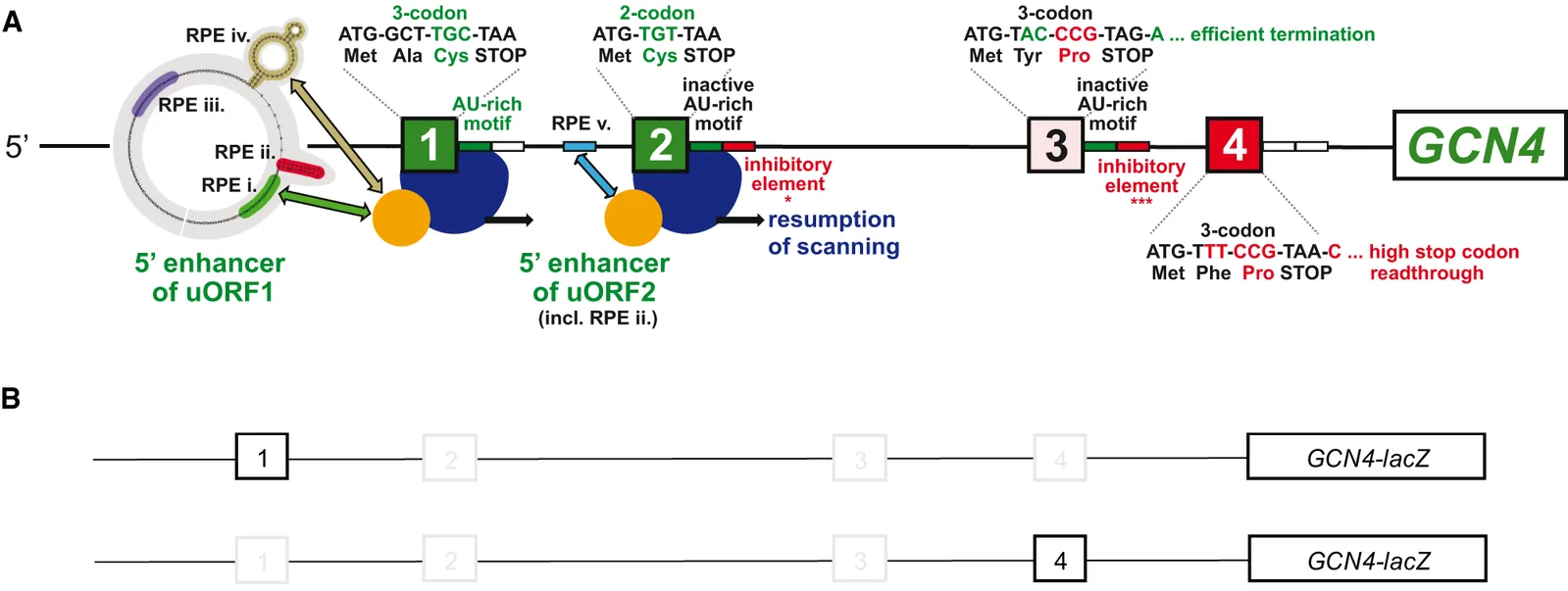
Summary of all *cis*-determinants that either promote or inhibit reinitiation on *GCN4* after translation of its four short uTranslons (uORFs). (*A*) Green color-coding indicates stimulatory effects of the corresponding *cis*-factors on efficiency of REI, whereas red color-coding indicates inhibitory effects (with the exception of RPE ii. of uORF1, which is also stimulatory); the number of asterisks below the inhibitory elements of the uORF2 and uORF3 3′ sequences illustrates the degree of their inhibition. Adapted with permission from Gunišová et al. (2016). (*B*) Schematic of the uORF1-only (*top*) and uORF4-only (*bottom*) constructs used in our reporter setup. uORFs with mutated TIS for each construct are grayed out.

Mechanistically, it has been determined that uORF1 and functionally similar uORF2 (Gunišová and Valášek 2014; Gunišová et al. 2016) are highly REI permissive due to the so-called REI-promoting elements (RPEs) forming the 5′ enhancer, which functional interacts with the eIF3a/TIF32 subunit of eIF3 (Fig. 1A; Grant et al. 1995; Szamecz et al. 2008; Munzarová et al. 2011; Gunišová and Valášek 2014). REI is also potentiated by sequences immediately following uORF1 and by penultimate codon identity of uORFs 1 and 2 (Grant and Hinnebusch 1994; Gunišová et al. 2016). In contrast, the REI-nonpermissiveness of uORF3 and uORF4 is given by both their codons identity and their 3′ nucleotide context (Fig. 1A). Note that REI efficiency of uORF4 is ∼3%–5% of that of uORF1 (Munzarová et al. 2011; Gunišová and Valášek 2014).

Building upon this knowledge, we used a well-established *GCN4*-based reporter system (Jendruchová et al. 2024) as a genetic tool to systematically search for new factors influencing efficiency of REI and ribosome recycling after translation of short uTranslons. In particular, we examined the REI rate after REI-permissive uORF1 and REI-nonpermissive uORF4 in yeast strains deleted for individual candidate factors compared to wild-type. This robust screening identified: (i) the decapping endonuclease Rai1p, and (ii) the Ssz1p/Zuo1p heterodimer, which forms the ribosome associated complex (RAC). Besides decapping, Rai1 (DXO in mammals) has a role in mRNA surveillance and ribosome biogenesis, among others (Xue et al. 2000; Fang et al. 2005; Doamekpor et al. 2020). The RAC complex is known to associate with ribosomes (Gautschi et al. 2001; Leidig et al. 2013; Lee et al. 2016; Kišonaitė et al. 2023) and controls nascent peptide folding (Kišonaitė et al. 2023), -1 frameshift (Lee et al. 2016) and stop codon readthrough (SC-RT) (Rakwalska and Rospert 2004). Our genetic and biochemical analysis revealed that these factors modulate both *GCN4* translational control and general translation.

## RESULTS

### Rai1p and the RAC complex influence REI after translation of uORF1 and uORF4

To screen our preselected candidates (see below), we used reporter plasmids containing ∼600 nt long 5′UTR of the *GCN4* gene followed by ∼150 nt of its translon fused in-frame with the *LacZ* reporter gene. To create a single uORF1 or uORF4 reporter, the AUG start sites of other three uTranslons were eliminated by point mutations in given constructs. In particular, the “uORF1” construct (Grant et al. 1995) bears only REI-permissive uORF1, while the “uORF4” construct (Mueller and Hinnebusch 1986) carries just REI-nonpermissive uORF4 (Fig. 1B). This reporter system has been successfully used in the past to dissect the specifics of REI and ribosome recycling on *GCN4* mRNA, and thus to decipher its translational control (Mueller and Hinnebusch 1986; Grant and Hinnebusch 1994; Grant et al. 1995; Hinnebusch 2005; Szamecz et al. 2008; Munzarová et al. 2011; Gunišová and Valášek 2014; Gunišová et al. 2016; Jendruchová et al. 2024). Inferring from this wealth of knowledge, we applied the following logic in our study. It was shown that REI defects manifest as lower REI rates after translation of uORF1, resulting in lower “uORF1” reporter activity (Szamecz et al. 2008; Munzarová et al. 2011). Thus, mutations or gene deletion impairing REI should lower the “uORF1” reporter activity compared to WT, warranting further analysis. On the other hand, ribosome recycling defects generally manifest as higher REI activity after translation of uORF4 (using the “uORF4” reporter compared to WT), since lower rates of ribosome recycling allow more 40S post-termination ribosomes to remain mRNA-bound even after translation of REI-nonpermissive uORF4 (Jendruchová et al. 2024). We performed our screening experiments in the BY4741 genetic background, utilizing strains obtained from the Yeast Deletion Collection (Brachmann et al. 1998).

The rationale for selecting our candidate genes was as follows. We selected genes from different classes of translation-related factors that had already been implicated in promoting either REI or recycling based on in vitro and/or indirect evidence. In parallel, we picked factors associated with different translation processes whose connection to ribosome recycling or REI could be predicted based on their known or proposed cellular functions. Among others, the selected gene groups included initiation factors, termination and related factors, RNA helicases, ribosome and/or translation-associated ATPases or GTPase and related proteins, factors associated with *GCN4* regulation, and finally, numerous as-yet-uncharacterized genes encoding proteins that copurified with yeast ribosomes in proteomic screens, with our particular interest put on 12 proteins designated as translation-machinery-associated (TMA) (Fleischer et al. 2006). (The list of candidates that passed the initial screening process including their reporter activities is provided in Supplemental Table S1.) We aimed at factors that exhibited strong (ideally more than 50%) change in one of the reporter constructs with minimal (ideally no) change in the other; to examine the process of REI or recycling separately. All the reporters were tested in three biological replicates for each deletion strain.

Our thorough screening identified three top candidates that matched our most stringent criteria: Ssz1p and Zuo1p (together forming the RAC complex), and Rai1p for validation and further analysis. There was no significant difference in *GCN4-LacZ* expression after uORF1 translation in *ssz1*Δ and *zuo1*Δ deletions strains compared to WT (Fig. 2A); however, we found a significant >1.5-fold increase in the uORF4 reporter activity in both RAC deletion strains (Fig. 2B), suggesting a recycling defect. Even though Rai1p was not known to function in cytoplasmic translation, our reporter analysis showed a ∼50% reduction in the uORF1 reporter activity in the *rai1*Δ deletion strain (Fig. 2C). It also displayed an apparent, although not statistically significant in our experimental setup, drop in the uORF4 activity (Fig. 2D); that is, we recorded virtually the opposite phenotype to that seen with the RAC proteins with this particular uTranslon. Therefore, based on our logic, Rai1p most probably promotes REI. Given the REI-nonpermissive character of uORF4, the observed statistical insignificance is not surprising because the Rai1p stimulatory effect must be small at this uORF.

**FIGURE 2.**
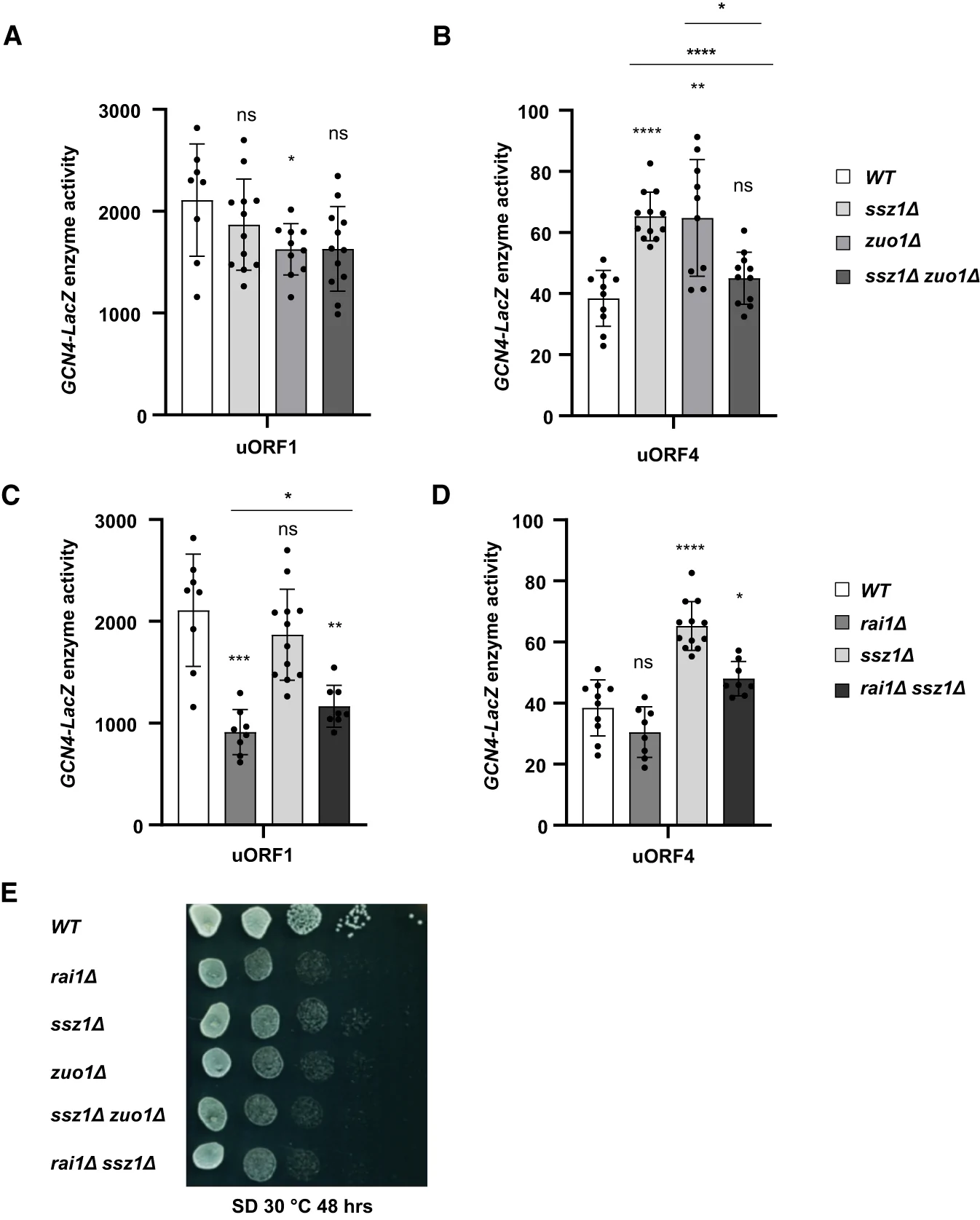
Impact of *rai1*, *ssz1*, and *zuo1* single and double deletion on *GCN4-lacZ* reporter expression and cell growth. (*A*) WT, *ssz1*Δ, *zuo1*Δ and *ssz1*Δ *zuo1*Δ yeast strains were transformed with a plasmid containing uORF1 followed by the *GCN4-LacZ* fusion. Reporter activity was assayed in at least five independent transformants and normalized as described in Materials and Methods. Data are presented as normalized mean values ± SD with points denoting each individual reporter value. Statistical analysis was performed using two-tailed unpaired Welch's *t*-test. (*) *P* < 0.05, (**) *P* < 0.01, (***) *P* < 0.001, (****) *P* < 0.0001, (ns) not significant. For source data of the measurements, please see Supplemental Data 1. (*B*) Same as in *A*, but the strains were transformed with the uORF4 reporter plasmid. (*C*,*D*) Same as in *A*,*B*, only WT, *rai1*Δ, *ssz1*Δ and *rai1*Δ *ssz1*Δ yeast strains were examined. (*E*) The indicated yeast strains were grown overnight, diluted to an OD_600_ = 0.5, then serially diluted (10x) and spotted onto SD plates. The plates were incubated at 30°C for 48 h.

Since Zuo1p and Ssz1p form a heterodimer, we created and tested a double deletion strain. Interestingly, loss of both RAC subunits led to the loss of the increased uORF4 phenotype with no impact on uORF1 (Fig. 2A,B). Informed by a yeast proteome survey reporting a physical interaction between Rai1p and Ssz1p (Gavin et al. 2006), we also created and tested the *rai1*Δ *ssz1*Δ double deletion strain. This strain exhibits a significant, nearly as robust decrease (almost twofold) in the uORF1 reporter expression as the single *rai1*Δ strain (Fig. 2C). Strikingly, *rai1*Δ *ssz1*Δ displays only modest (but still significant) increase in the uORF4 reporter expression, which is practically an average activity of the *rai1*Δ and *ssz1*Δ individual activities combined (Fig. 2D). These phenotypes suggest: (i) a dominant uORF1-associated REI phenotype of *rai1*Δ versus *ssz1*Δ; and (ii) a compensatory uORF4-associated REI phenotype of both single deletions. We failed to create a *rai1*Δ *zuo1*Δ strain despite many tries.

Collectively, loss of Rai1p hampers efficiency of REI after translation of uORF1 (and to some degree also after uORF4) with no effect exerted by the RAC complex at this uTranslon, whereas the loss of RAC complex impairs ribosome recycling at uORF4 (thus allowing increased REI past this uTranslon), which can be nearly fully rescued by a concomitant loss of Rai1p. These findings might point to a possible physical interaction between Raip1 and the RAC components (see below).

### Rai1p and the RAC complex modulate *GCN4* translational control

Next, we analyzed the growth phenotype of WT and all deletions and their combinations using the yeast spot assay (Fig. 2E). All single deletion strains exhibited a slow growth phenotype (Slg^−^) at 30°C on SD media, underscoring the importance of these factors for cell growth and fitness. The phenotype was more severe in the case of *rai1*Δ and *zuo1*Δ compared to *ssz1*Δ, but no additive or compensatory phenotypes were observed (Fig. 2E).

The key experiment was to test whether deletions of *rai1*Δ, *zuo1*Δ, and *ssz1*Δ perturb general translational control of *GCN4* under amino acid stress conditions induced by 3-aminotriazole (3-AT), because only if they do, their REI-associated role in the cell is physiologically significant. 3-AT functions as a competitive inhibitor to imidazole glycerol-phosphate dehydratase (encoded by the *HIS3* gene), which acts at the last step of the histidine biosynthesis pathway (Klopotowski and Wiater 1965; Alifano et al. 1996). Lack of histidine leads to accumulation of uncharged histidyl-tRNAs, which activates the Gcn2p kinase that phosphorylates eIF2α, which in turn induces Gcn4p translation (Dever et al. 1992; Wek et al. 1995). Since the BY4741 genetic background bears *his3*Δ (Brachmann et al. 1998), a functional *HIS3* gene on an empty shuttling plasmid needed to be introduced to all tested strains along with our well-established reporter plasmid (B196). It contains only uORF1 and uORF4 in the *GCN4* 5′UTR (Fig. 3A) and was shown to faithfully recapitulate the complex *GCN4* translational control (Mueller and Hinnebusch 1986).

**FIGURE 3.**
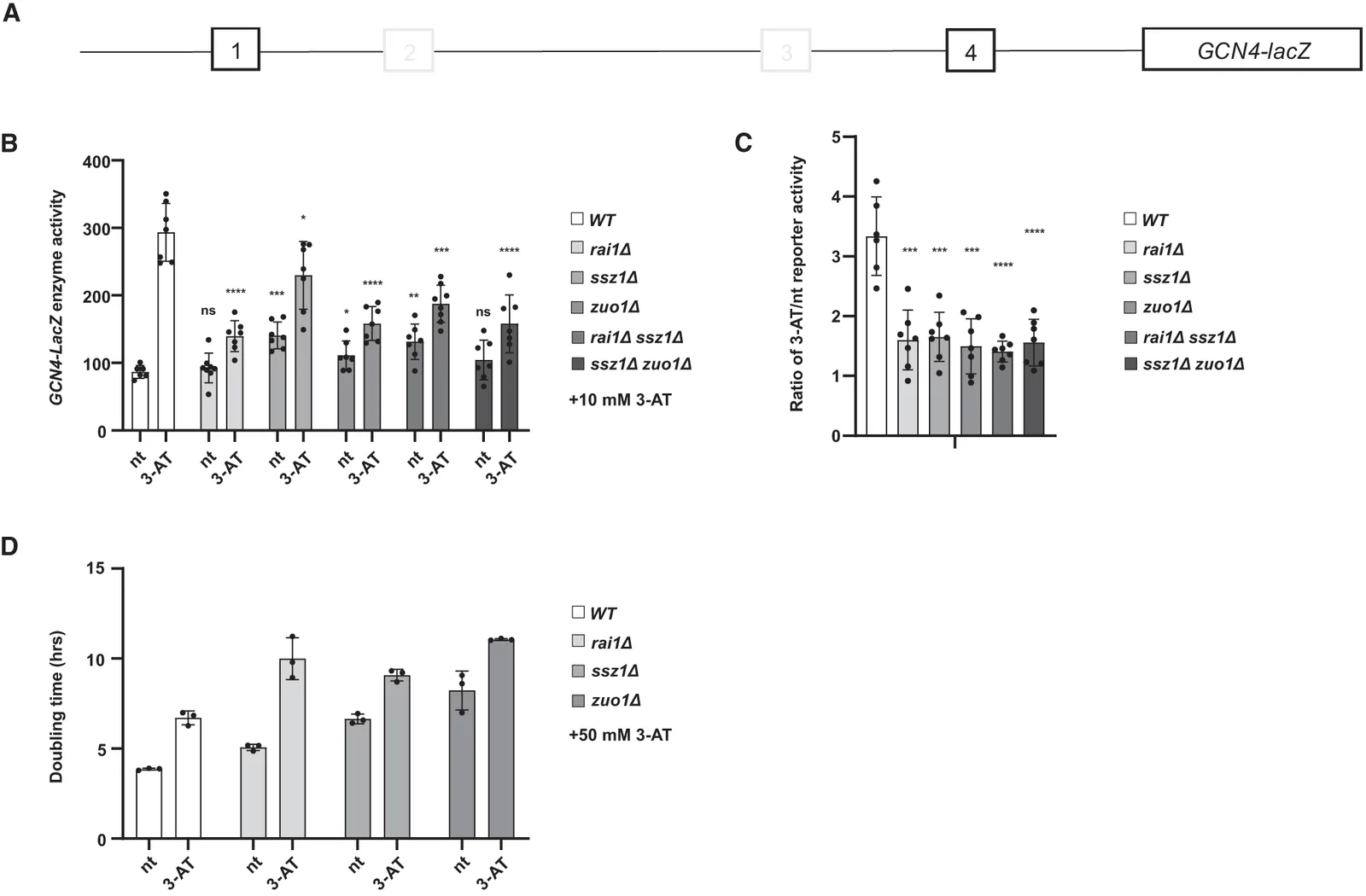
Rai1p and the RAC complex modulate *GCN4* translational control. (*A*) Schematic of the uORF1 + uORF4 constructs used in our reporter setup. uORFs with mutated TIS for each construct are grayed out. (*B*) The indicated yeast strains were cotransformed with a vector containing the *HIS3* gene and another one containing the uORF1 + uORF4 reporter, and the resulting transformants were grown in parallel with or without 10 mM 3-AT for 4 h. Reporter activity was assayed in at least five independent transformants. Data are presented as normalized mean values ± SD with points denoting each individual reporter value. Statistical analysis was performed between WT versus deletion strains for untreated and treated values, respectively, using two-tailed unpaired Welch's *t*-test. (*) *P* < 0.05, (**) *P* < 0.01, (***) *P* < 0.001, (****) *P* < 0.0001, (ns) not significant. For source data of the measurements, please see Supplemental Data 1. (*C*) Ratios of reporter activities from (A) between 3-AT-treated and untreated cultures, indicating the rate of the Gcn4p induction in each strain. For the statistical analysis, ratios were normalized to log_2_ values. Statistical analysis of WT log_2_-ratios versus deletion strain log_2_-ratios was carried out using two-tailed unpaired Welch's *t*-test. (*) *P* < 0.05, (**) *P* < 0.01, (***) *P* < 0.001, (****) *P* < 0.0001, (ns) not significant. Log_2_-transformed ratios can be found plotted in Supplemental Figure S1A. (*D*) Doubling time of WT, *rai1*Δ, *ssz1*Δ and *zuo1*Δ strains transformed with a plasmid containing *HIS3* grown in minimal SD media. Values are averages of three biological replicates with individual values indicated by data points.

The β-galactosidase reporter assays using this specific reporter plasmid expressed in WT and all deletion strains incubated with or without 10 mM 3-AT revealed the following effects (Fig. 3B): (i) no significant changes were seen under normal conditions in *rai1*Δ and *ssz1*Δ *zuo1*Δ, while *ssz1*Δ, *zuo1*Δ and the *rai1*Δ *ssz1*Δ double deletion strain exhibited slightly elevated reporter expression (clearly, *ssz1*Δ seems to be the major driver of this effect); (ii) all deletions or their combinations robustly reduced the *GCN4* inducibility under starvation compared to WT; (iii) the compensatory uORF4-associated REI phenotype of *rai1*Δ *ssz1*Δ did not translate into the overall GCN4 control (highly likely due to the dominant uORF1-associated REI phenotype of *rai1*Δ vs. *ssz1*Δ). The ratio of 3-AT-treated/untreated reporter activities in each strain can be seen in Figure 3C and is practically identical for all deletion strains. To correct for the inherent asymmetry between fold-increases and fold-decreases in ratio data (e.g., 2.0 and 0.5 ratios represent the same twofold change), the ratio values were transformed to log_2_-ratio values for the statistical analysis of WT versus deletion strain ratios. These log_2_-transformed values are plotted in Supplemental Figure S1A and given in Supplemental Data S1. As expected, the ratios of all deletion strains were significantly lower than the WT ratio. As a negative control, we used a protein identified in our screening as a nonspecific hit. In particular, the *WHI4* gene deletion had neither specific (a significant change in one of the reporter constructs with no or only minimal change in the other) nor particularly significant effect on any of our constructs with a single uORF (Supplemental Fig. S1B). The induction of the uORF1-4 construct using 3-AT was also unaffected (Supplemental Fig. S1C). These results clearly demonstrate that the disruption of Gcn4p induction is specific to our mutants and is not caused by the activity of any factor unrelated to the *GCN4* translational control. As expected, the 3-AT treatment induced phosphorylation of eIF2α-Ser51 in WT and all deletion strains (Supplemental Fig. S1D). Interestingly, all deletion strains displayed slightly elevated basal levels of phosphorylation compared to WT, mainly *ssz1*Δ and *zuo1*Δ. Theoretically, this phenomenon could be attributed to perturbed nascent peptide folding or biogenesis. Nonetheless, since essentially the same effect was seen in control *whi4*Δ, which does not impact stress-induced *GCN4* upregulation, and given the markedly different basal levels of *GCN4* reporter expression between *ssz1*Δ and *zuo1*Δ (Fig. 3B), which are not reflected in the extent of their basal eIF2α-Ser51 phosphorylation levels, we do not think that this basal level variability significantly contributes to the observed impairment of *GCN4* inducibility in the *ssz1*Δ, *zuo1*Δ, and *rai1*Δ strains.

Since all tested deletion strains reduced the *GCN4* inducibility by at least ∼50% (Fig. 3C), we next examined whether the low inducibility translates also into a physiologically relevant growth defect. To do that, WT and individual deletion strains transformed with the *HIS3* empty plasmid were assayed for growth rates in the liquid minimal (SD) media supplemented with leucine, methionine and uracil, either supplemented with 50 mM 3-AT or not. Their doubling time (dt) was calculated, and we compared the fold-change between WT and deletion strains for untreated and 3-AT-treated cultures (Fig. 3D). Under normal conditions, the dt of *rai1* deletion was least affected compared to wt, followed by *ssz1* and *zuo1* deletions, while under stress conditions, the dt of all the deletions was comparable (∼1.5-fold higher than wt). Thus, the markedly reduced *GCN4* inducibility resulted in a comparably severe slow growth of all mutants, despite the fact that their starting position (dt under nt) differed. Collectively, our findings illustrate the importance of all three factors in the physiological well-being of the yeast cells, which can be at least partially attributed to the proper functioning of the delayed REI mechanism of the *GCN4* regulation.

### Rai1p associates with 40S subunits and actively translating 80S ribosomes

To assess the impact of single or double deletions of *RAI1*, *SSZ1*, and *ZUO1* on general translation rates, we performed polysome profiling using cycloheximide (CHX), which freezes elongating ribosomes (Fig. 4). As shown in Figure 4B, *rai1*Δ exhibited the so-called “halfmers” that can be seen as trailing shoulders of 80S monosomes and polysomes (marked with an arrow) and suggests either a 60S biogenesis defect or 60S–48S subunit joining defect. At the same time, *rai1*Δ, exhibited a mild polysome runoff (indicated by reduction in polysomal fractions) (Fig. 4B). A more pronounced runoff was seen with *ssz1*Δ and *zuo1*Δ single and *ssz1*Δ *zuo1*Δ and *rai1*Δ *ssz1*Δ double deletions, indicating a clear reduction of initiation rates (Fig. 4C–F). Interestingly, as in the case of the “uORF4 phenotype” of *ssz1*Δ that was suppressed by *rai1*Δ (Fig. 2D), here, combining *rai1*Δ and *ssz1*Δ suppressed the halfmer phenotype of *rai1*Δ.

**FIGURE 4.**
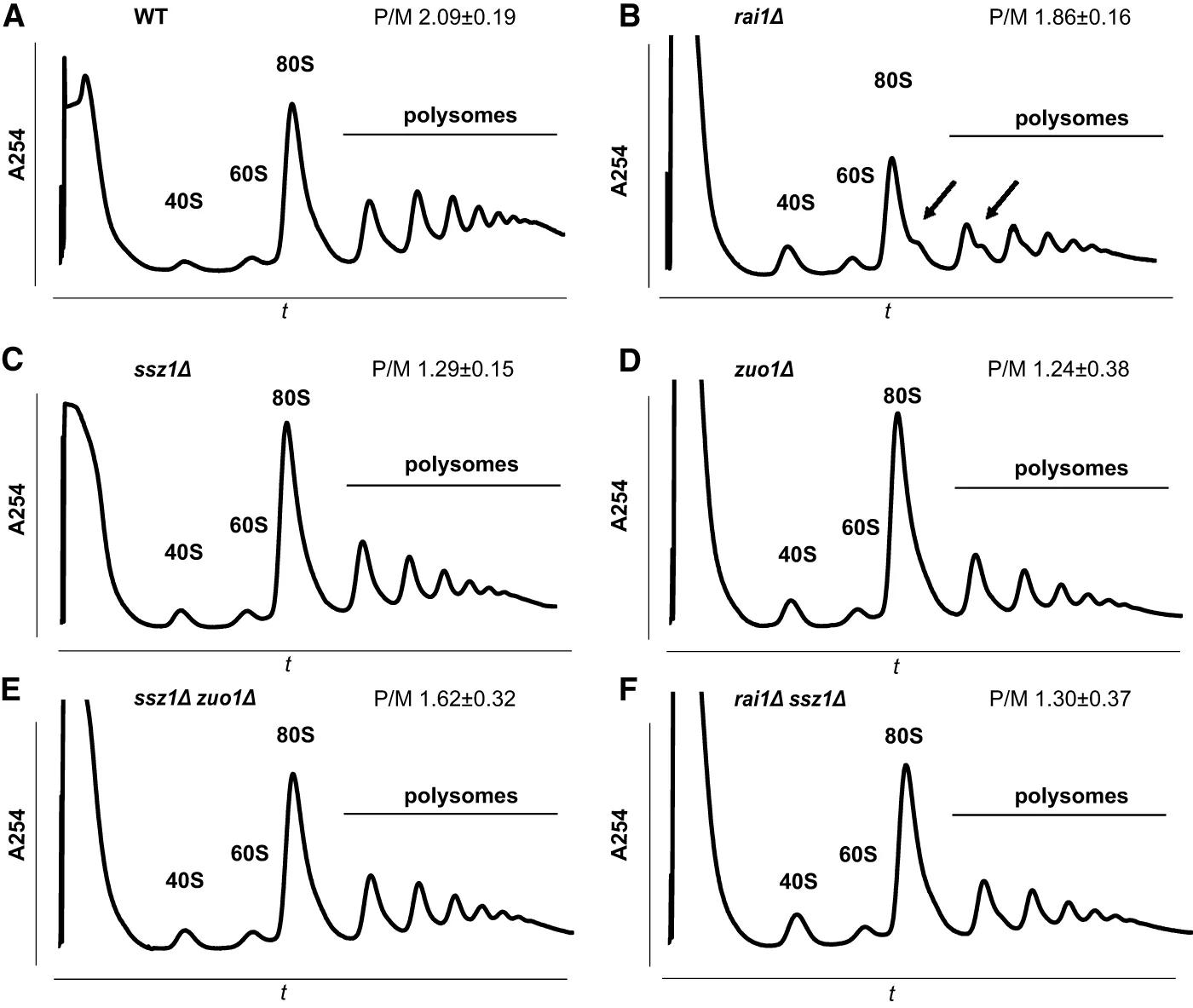
Polysome profiles of wild type and indicated deletion strains. (*A*–*F*) Yeast strains were grown to an OD_600_ ∼0.8 in YPD media, treated with cycloheximide and WCEs were prepared. WCE aliquots containing 8U of total RNA were layered onto 5%–50% sucrose gradients and separated by ultracentrifugation at 260,000*g* for 2.5 h. Gradients were scanned at 254 nm to visualize ribosomal species. Positions of 40S, 60S, 80S and polysomal species, as well as halfmers, are indicated, and P/M ratios are displayed in the upper right corner.

Next, we wished to examine whether our three candidates can reciprocally suppress Slg^−^ phenotypes associated with their deletions when in high copy. Therefore, we created overexpression plasmids carrying the *RAI1*, *ZUO1*, and *SSZ1* genes fused C-terminally with a 6× Gly linker followed by 3× FLAG tag. These protein fusions were flanked by 500 nt of the natural 5′UTR and 3′UTR contexts of their corresponding genes. Growth complementation spot assays confirmed that the FLAG-tagged fusion proteins complemented growth defects in the corresponding deletion strains (Supplemental Fig. S2A–C). However, we saw no growth complementation of *rai1*Δ with either *SSZ1-FLAG* or *ZUO1-FLAG* (Supplemental Fig. S2B); of *ssz1*Δ with either *RAI1-FLAG* or *ZUO1-FLAG* (Supplemental Fig. S2B); and of *zuo1*Δ with either *SSZ1-FLAG1* or *RAI1-FLAG* (Supplemental Fig. S2C).

Given the observed REI/recycling phenotypes, we were curious to test whether these three proteins associate with small ribosomal subunits, as could be expected from our results and some previous studies; the Ssz1p:Zuo1p heterodimer has been shown to occur on ribosomes (Gautschi et al. 2001; Leidig et al. 2013; Lee et al. 2016; Chen et al. 2022). However, nothing is known about cytoplasmic Rai1p and ribosomes. Thus, we grew *rai1*Δ, *ssz1*Δ, and *zuo1*Δ strains transformed with the corresponding *RAI1/SSZ1/ZUO1-FLAG* plasmids, fixed the cells with 1% formaldehyde (FA) as a crosslinking agent, generated and separated WCEs by high velocity centrifugation, collected fractions from sucrose gradients and performed western blotting to determine their presence in each fraction (Fig. 5). The eIF3a/Tif32p subunit of eIF3, known to associate with initiating, elongating and terminating ribosomes (Valásek et al. 1998, 2003; Beznosková et al. 2013, 2015; Mohammad et al. 2017; Wagner et al. 2020), was used as a control. While Rai1-FLAG was detected mainly in the top (ribosome-free) fractions, a sizeable proportion co-sedimented in the 40S-containing fractions, as well as in monosomal and even polysomal fractions, containing actively translating ribosomes (Fig. 5A). The Ssz1-FLAG and Zuo1-FLAG showed a very similar co-sedimentation pattern to Rai1p (Fig. 5B,C), consistent with the published studies (Gautschi et al. 2001; Leidig et al. 2013; Lee et al. 2016; Chen et al. 2022). To further verify the binding of Rai1p to ribosomes, we compared, as one condition, cross-linking with 1% FA and, as a second condition, treatment with CHX (which halts ribosomal elongation but does not form cross-links between them). We then loaded the samples onto 7%–30% gradients to closely monitor the top and 40S-containing fractions only. We performed immunoblots of the collected fractions for our protein of interest and for the control protein PGK1—a ubiquitously expressed cytoplasmic gene encoding 3-phosphoglycerate kinase, which should not be bound to the ribosomes (Supplemental Fig. S3A,B). In samples treated with both CHX and FA, we did indeed observe Rai1p-FLAG binding to the 40S, but not PGK1 binding. As expected, this difference was more pronounced in the case of FA, which is known to stabilize factors that bind to the 40S; unlike Rai1p-FLAG, PGK1 showed no difference between these two conditions.

**FIGURE 5.**
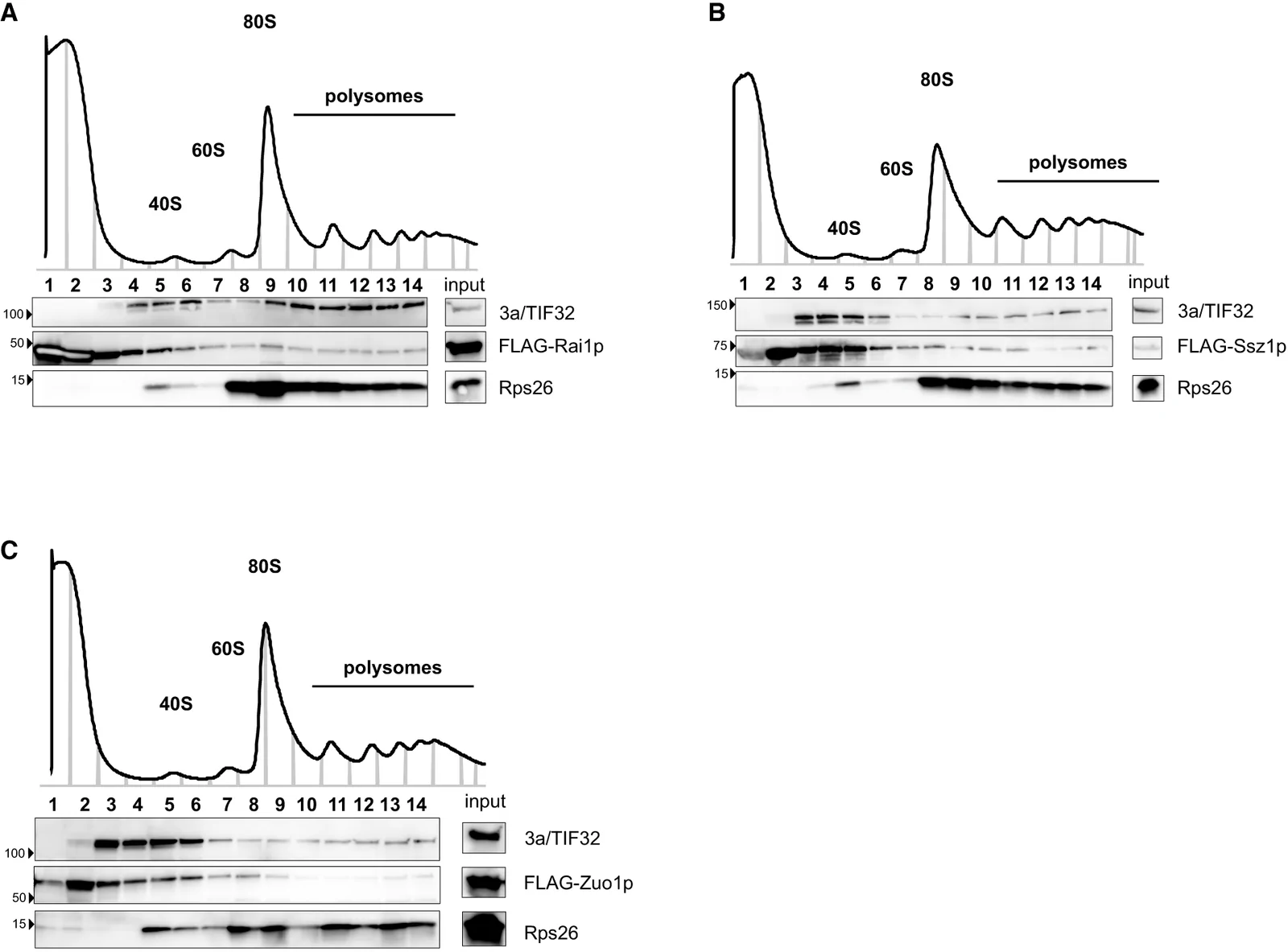
Rai1p associates with 40S subunits and actively translating 80S ribosomes. (*A*) The *rai1*Δ strain transformed with YEplac181 carrying a *RAI1-FLAG* fusion was grown in SD media to an OD_600_ ∼0.9–1.0 and crosslinked with 1% formaldehyde; YEplac was used because YCplac-driven expression was poor. 20 U of A_260_ were layered onto 5%–50% sucrose gradients and separated by ultracentrifugation at 39,000rmp for 2.5 h. Gradients were scanned at 254 nm to visualize ribosomal species, fractions were collected, precipitated with 96% EtOH, and subjected to western blot analysis. Fraction numbers are indicated above the immunoblot; see Materials and Methods for details. (*B*) Same as *A*, except that *ssz1*Δ transformed with YCplac111 carrying a *SSZ1-FLAG* fusion was examined. (*C*) Same as *A*, except that *zuo1*Δ transformed with YCplac111 carrying a *ZUO1-FLAG* fusion was examined.

### Rai1p and Ssz1p interactomes

To explore an interactome of Rai1p as a newly characterized translation factor, we performed coimmunoprecipitation experiments (co-IP) of Rai1-FLAG-associated complexes. As a reference co-IP sample, we used Ssz1-FLAG-associated complexes, and as negative control, we used the *rai1*Δ or *ssz1*Δ deletion strains. The yeast cultures transformed with YEplac181 plasmids carrying FLAG-tagged fusions were grown to an OD_600_ ∼ 0.8 in minimal SD media, cells were lysed and immunoprecipitated complexes were then sent for liquid chromatography/mass spectrometry (LC/MS) analysis (Fig. 6A,B; source data can be found in Supplemental Data S2). Due to high abundance of ribosomal proteins in cells in general, which can potentially obfuscate the MS analysis (Mellacheruvu et al. 2013), we performed co-IP experiments under more stringent salt conditions. This way we could distinguish background contaminants from true interactors (see the Materials and Methods section for more details). Validating our co-IP, Rai1p pulled down Rat1p, its canonical nuclear interaction partner (Xue et al. 2000). Among the most intriguing Rai1p-specific hits was Dbp5p, which is a DEAD-box helicase that has been implicated in RNA metabolism, translation termination and RNA quality control (for review, see Querl and Krebber 2023). In addition, ribosomal proteins were expectedly detected, as well as several proteins from amino acid biosynthetic pathways, such as Arg1p, His4p, Hom2p and Cpa2p, which was not expected (Fig. 6A). Validating our approach further, Ssz1-FLAG brought down its RAC partner—Zuo1p—as one of the top hits (Fig. 6B). Furthermore, expected enrichment of ribosomal proteins was seen including the 40S head interacting hub—Asc1p, plus other ribosome or ribosome biogenesis-associated factors (Utp11/20p, Bcp1p, Spb1p), and some proteins involved in amino acid synthetic pathways (Asn1/2p, Lys1p, Aat1p) (Fig. 6B). Interestingly, both co-IP experiments yielded Snz1p, a pyridoxine biosynthesis protein whose gene belongs among Gcn4p transcription factor target genes (Fig. 6A,B; Qiu et al. 2004). Since we saw no Rai1p in the Ssz1p-FLAG co-IP and vice versa, we tested their prospective interaction using an in vitro protein–protein binding assay. Indeed, we observed a binding between GST-Ssz1p and radiolabeled Rai1p, which was weaker than the binding with its well-known interaction partner Zuo1p but still obvious (Fig. 6C). Despite the fact that none of the identified hits clearly pointed to a specific role for the studied proteins in the REI mechanism and/or ribosomal recycling, overall, our interactome data further support Rai1p's newly described role in translation as a ribosome associated factor.

**FIGURE 6.**
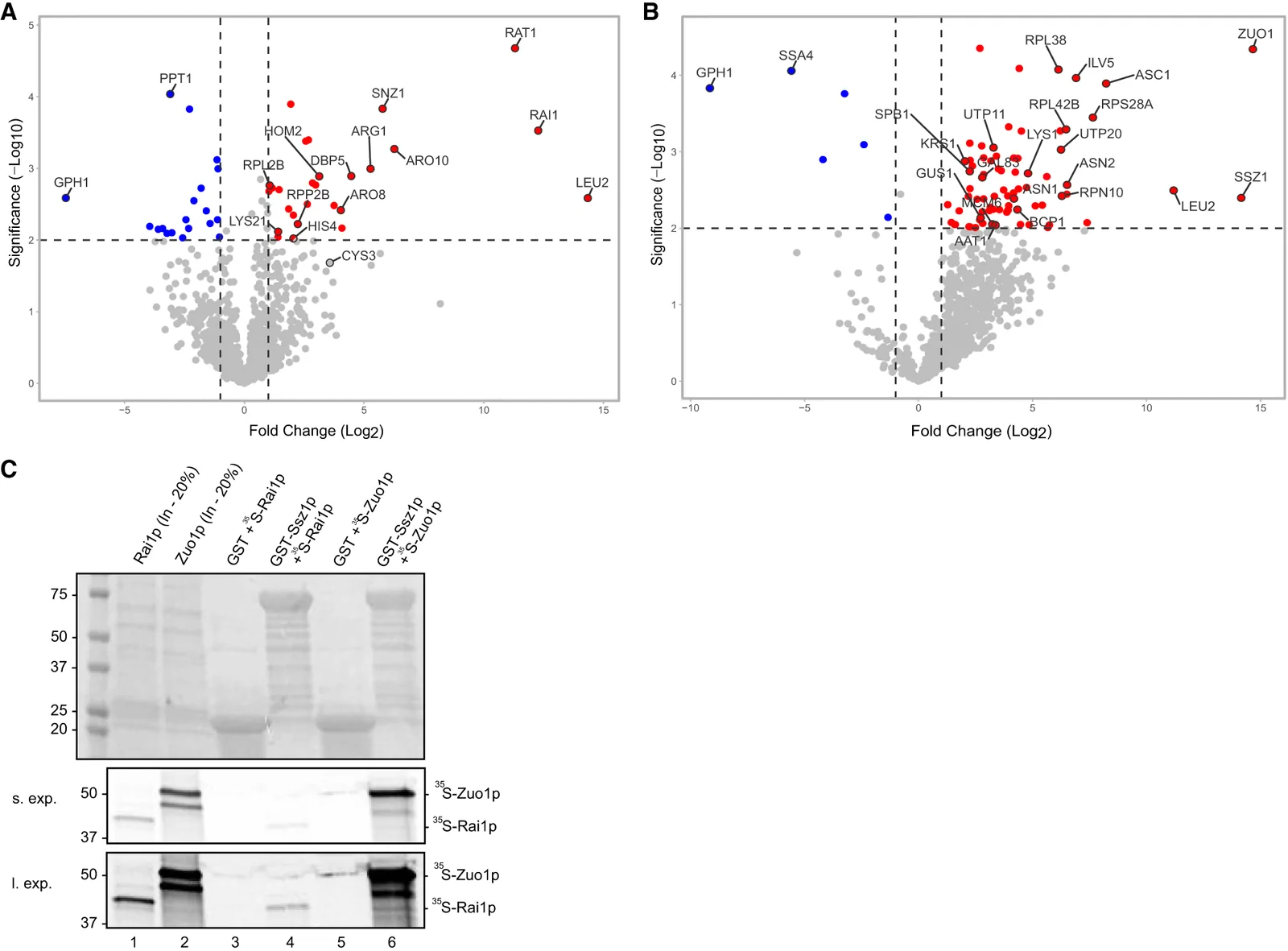
Rai1p and Ssz1p interactomes. (*A*,*B*) Volcano plots of LC/MS analysis of complexes pulled down by (*A*) *RAI1-FLAG* expressed in *rai1*Δ and (*B*) *SSZ1-FLAG* in *rai1*Δ. As negative controls we used deletion strains. Log_2_FC threshold was set as −1/+1 (corresponding to twofold change) and −Log *P*-value threshold was set as 2 (corresponding to *P* = 0.01). (*C*) Ssz1p fused to GST (lanes *4* and *6*) or GST alone (lanes *3* and *5*) was expressed in *E. coli*, immobilized on glutathione-Sepharose beads, and incubated with the ^35^S-labeled Rai1p (lanes *3* and *4*) and Zuo1p (lanes *5* and *6*) at 4°C for 2 h. The beads were washed three times with phosphate-buffered saline, and bound proteins were separated by SDS-PAGE. Gels were first stained with GelCode Blue Stain Reagent (*top* panel) followed by autoradiography (*bottom* panels are the same but differ in exposition time). Lanes *1* and *2* show 20% of the input (In) amounts of ^35^S-labeled Rai1p and Zuo1p, respectively.

## DISCUSSION

In our long-standing effort to identify new *cis-* or *trans-*acting factors contributing to REI, we employed a *GCN4*-based reporter system and identified three new factors modulating efficiency of REI at short uTranslons—Rai1p, Ssz1p and Zuo1p. The Ssz1p/Zuo1p heterodimer forming the RAC complex has already been implicated in translational control—specifically, it influences cotranslational folding (Gautschi et al. 2001; Huang et al. 2005; Zhang et al. 2020b; Kišonaitė et al. 2023), -1 frameshifting (Muldoon-Jacobs and Dinman 2006) and SC-RT (Rakwalska and Rospert 2004). The mammalian RAC complex (Hsp70L1/Ssz1 and MPP11/Zuo1), shown to complement the yeast RAC deletion, similarly modulates SC-RT (Jaiswal et al. 2011). We propose that the higher uORF4-only reporter activity that we observed with the *ssz1*Δ and *zuo1*Δ single deletions strains (Fig. 2B) most probably suggests a ribosome recycling defect. First, it has a similar phenotype to deletions in specific *TMA* genes whose protein products promote 40S subunit recycling, as we and others have previously reported (Mohammad et al. 2017, 2021; Szamecz et al. 2008; Young et al. 2018, 2021; Bohlen et al. 2020b; Jendruchová et al. 2024). Second, uORF1 is highly REI permissive thanks to RPEs that functionally interact with the eIF3a/TIF32 subunit of eIF3 (Grant et al. 1995; Szamecz et al. 2008; Munzarová et al. 2011; Gunišová and Valášek 2014) to prevent full ribosome recycling. As such, mutations impairing ribosome recycling should show no phenotype with the uORF1 only construct, as was observed (Fig. 2A). Third, the effect of dysregulated −1 or +1 frameshifting can be ruled out, because there are several stop codons in all frames between the main *GCN4* translon and uORF4. Finally, SC-RT can also be discounted because heightened SC-RT decreases and not increases the efficiency of REI past uORF4 (Gunišová et al. 2016).

Somewhat unexpectedly, combining *ssz1*Δ and *zuo1*Δ did not further increase the uORF4 reporter activity but virtually nullified the uORF4-specific effect (Fig. 2B). This result indicates that these two factors do not act in a cooperative manner during recycling, even though they can form a heterodimer. The simplest explanation is that each protein independently contributes to controlling a different recycling step. However, to complete the recycling process, the clearance of both independent steps is required, regardless of the order in which it occurs. For example, binding of Ssz1p and Zuo1p to the post-termination ribosome could be mutually exclusive and, therefore, only after the Ssz1p task is completed and Ssz1p leaves, Zuo1p can bind to perform its own task, and vice versa. It does not matter which one is done first, both must be performed to avoid blocking the whole process, but they cannot be performed simultaneously. Deleting both factors may modestly reduce the overall recycling rate (neither of them is essential), but at the same time it will remove processivity blocks.

Rai1p has been implicated in mRNA degradation (Das et al. 2003), transcription termination (Kim et al. 2004; El Hage et al. 2008; Park et al. 2015), mRNA surveillance and decapping (Wang et al. 2015; Zhang et al. 2020a), and rRNA processing (Sydorskyy et al. 2003; Fang et al. 2005). However, these functions are predominantly bound to the nucleus, often in cooperation with its nuclear binding partner Rat1p. Interestingly, the *rat1* loss-of-function mutation prompts Rai1p to nearly completely relocalize to the cytoplasm (Sydorskyy et al. 2003), where its roles have not been explored so far. Here, deletion of *rai1* manifested ∼50% decrease in the uORF1-only reporter activity (Fig. 2C), suggesting that one of its cytoplasmic roles could be in promoting REI. The extent of this effect is remarkably similar to those displayed by mutations of two subunits of the eIF3 complex. The *tif35-KLF* mutation of eIF3g reduced the uORF1-only reporter activity down to 52% (Cuchalová et al. 2010), and the selected eIF3a mutations reduced it to ∼30%–50% (Munzarová et al. 2011). It is important to emphasize that although it is possible that *rai1*Δ could affect the stability of our reporter mRNAs, as it was suggested to promote mRNA degradation (Das et al. 2003), this potential issue is eliminated by our normalization settings (see Materials and Methods). Rai1p and Ssz1p were shown to interact (Gavin et al. 2006). Interestingly, while combining *ssz1*Δ with *rai1*Δ had no impact on the significantly reduced ORF1 reporter activity observed in the single *rai1*Δ deletion, it produced a compensatory phenotype at uORF4—increased uORF4 activity of *ssz1*Δ and decreased activity of *rai1*Δ met in the middle and reached nearly wild type values (Fig. 2C,D). This is consistent with the “dominant” REI permissive phenotype of uORF1, where only a defect in REI can manifest itself, but not in recycling, as explained above. However, at uORF4, a defect in REI that further reduces the residual REI capacity of uORF4 may be compensated for by a defect in 40S subunit recycling.

Most importantly, using a simplified uORF1 + uORF4 reporter system and growth assays, we demonstrated that absence of each of these factors diminished the natural inducibility of *GCN4* translation under starvation conditions (by at least 50%) (Fig. 3A,B), which resulted in a dramatic slow growth phenotype (Fig. 3C). Although the precise molecular mechanism of their action is unclear, we posit that Rai1p contributes to the overall control by promoting efficient REI after uORF1, while Ssz1p and Zuo1p independently ensure complete ribosome recycling after uORF4 translation (Fig. 7). Both of these events are critical for the entire system to operate properly. Interestingly, Ssz1p/Zuo1p have already been implicated in regulation of various stress responses through differing mechanisms, including mito-protein induced stress and heat stress (Qian et al. 2026), TOR signaling perturbation (Gillies et al. 2012; Black et al. 2023), arsenite stress (Rodrigues et al. 2023), cell wall integrity pathway (Gillies et al. 2012) or pleiotropic drug resistance (Eisenman and Craig 2004; Prunuske et al. 2012).

**FIGURE 7.**
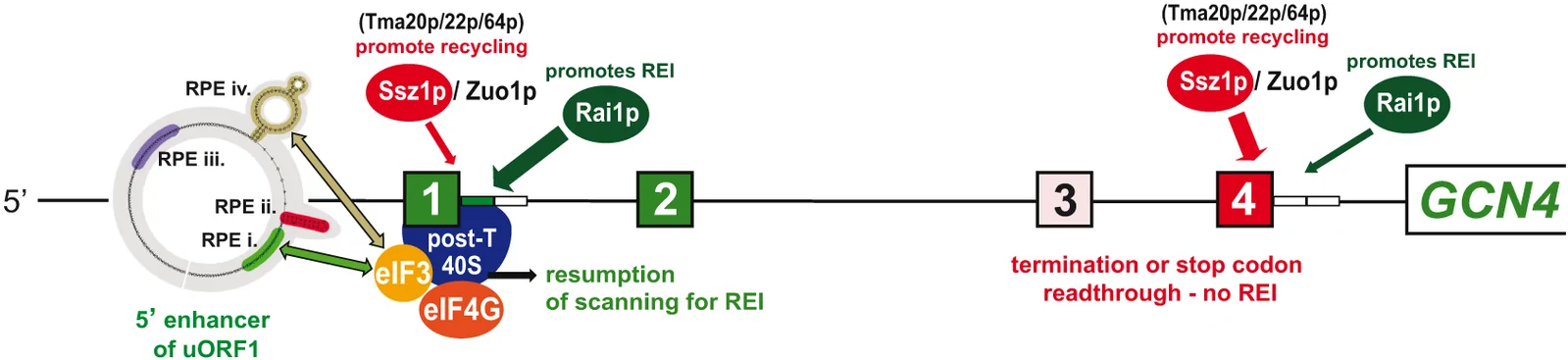
The proposed mechanism of action of Rai1p, Zuo1p, and Ssz1p in *GCN4* translational control. Raip1 contributes to overall control by promoting efficient REI after uORF1, alongside eIF3 and eIF4G, while Ssz1p and Zuo1p independently ensure complete ribosome recycling after uORF4 translation, alongside Tma20p/22p/64p (Szamecz et al. 2008; Mohammad et al. 2017, 2021; Young et al. 2018, 2021; Bohlen et al. 2020b; Jendruchová et al. 2024).

We found that Rai1p-FLAG mostly associated with free 40S subunits, and less with 80S monosomes and translating polysomes (Fig. 5A; Supplemental Fig. S3), which is partially at odds with published results (Sydorskyy et al. 2003), suggesting that Rai1p binds predominantly to pre-60S particles during 60S biogenesis. However, the authors used cycloheximide to freeze translating ribosomes and admitted that binding to other ribosomal species might occur, but it is below the detection limit of their setup (Sydorskyy et al. 2003). Our approach was based on crosslinking cells with 1% formaldehyde, which allowed us to detect more transient interactions. Based on our results, we therefore propose that binding of Rai1p to pre-60S does not preclude Rai1p from remaining bound or rebinding to mature ribosomes and thus contributing to translational control.

Our findings combined with the aforementioned fact that Rai1p almost completely relocalizes to the cytoplasm in the *rat1* mutant cells may have interesting implications. It was proposed that Rat1p actively keeps Rai1p tethered to the nucleus to aid in biogenesis. When inactive, Rai1p may migrate to the cytoplasm while bound to immature 60S subunits (Sydorskyy et al. 2003). Upon amino acid starvation, rRNA transcription is reportedly depleted by ∼80% to downregulate ribosome biogenesis and conserve metabolic energy (Conesa et al. 2005). This rapid depletion could reduce Rat1p activity and thus allow partial translocation of Rai1p to the cytoplasm to contribute to *GCN4* translational control under stress.

Our stringent co-IP analyses using FLAG-tagged Rai1p as bait followed by LC/MS analysis identified Dbp5p as a major hit for Rai1p, in addition to several ribosomal proteins (Fig. 6A,B). Interestingly, among other hits, several enzymes of amino acid biosynthetic pathways were also recorded. Virtually the same applies to Ssz1p-FLAG LC/MS. The physiological significance of these findings is unclear and is currently under investigation. While Rai1p-FLAG did not pull down Ssz1p and vice versa, we detected a direct interaction between these two proteins in vitro (Fig. 6C), consistent with a previous proteome survey report (Gavin et al. 2006). Given that Rai1p interaction with Zuo1p has also been seen (Michaelis et al. 2023), we propose that Rai1p probably coexists with each of its binding partners on the ribosome, but not simultaneously, where they exert antagonistic activities—stimulating REI versus ribosome recycling.

In summary, we have identified three new factors contributing to *GCN4* translational control in the opposite manner. These findings further expand the arsenal of *cis-* and *trans-*acting factors (summarized in Fig. 7) that have been implicated in this delicate, exemplary and intensively studied mechanism of delayed reinitiation so far. It will be interesting to examine whether their human homologs contribute to *ATF4* translational control in a similar fashion.

## MATERIALS AND METHODS

### β-Galactosidase reporter assays

β-Galactosidase activities were assayed in whole cell extracts (WCEs) as described (Jendruchová et al. 2024). Briefly, cells were transformed with plasmids bearing *GCN4-lacZ* fusion reporters (along with a *HIS3* carrying shuttle vector for Fig. 3) and grown in synthetic SD media supplemented with histidine, leucine and methionine (for Fig. 3 only leucine and methionine) to mid-log phase. Cells were centrifuged, washed with deionized water and broken using glass beads in Breaking Buffer (100 mM Tris 8.0, 4% Glycerol, 1 mM β-mercaptoethanol), then clarified by centrifugation (18,400*g*/30 min/4°C). β-Galactosidase activity (measured as nanomoles of o-nitrophenyl-3-d-galactose cleaved per minute per milligram of protein) was normalized to protein concentration of the WCE, which was measured by Bradford assay with BSA as standard.

The normalized β-galactosidase activity for each reporter construct in transformants of deletion strains was determined by dividing the unnormalized β-galactosidase activities by a normalization factor. This factor was calculated separately for each individual experiment by assaying the activity of the “uORF-less” reporter (p227) in WT and deletion strains in four transformants under the same conditions as uORF1-only/uORF4-only reporters. Then, the mean of each deletion strain “uORF-less” activities was divided by the mean of WT “uORF-less” activities, determining the normalization factor.

### Plasmids and yeast strains

All plasmids and primers used are listed in Supplemental Tables S2 and S3. Cloning was carried out in the DH5α *Escherichia coli* strain.

The plasmids were constructed as follows: pKJ64 was constructed by replacing the *PstI-BamHI* fragment of YEplac181 by synthesized DNA fragment 1 (GeneString). pKJ65 was constructed by replacing the *HindIII-EcoRI* fragment of YEplac181 by synthesized DNA fragment 2. pKJ72 and pKJ73 were generated by subcloning the synthesized fragments from pKJ64 and pKJ65 into the YCplac111 backbone, as the multicloning site in YEplac181 and YCplac111 is identical. pKJ80 was constructed in two steps: first, replacing the *PstI-BamHI* fragment of YEplac181 by a fragment amplified from yeast cDNA using primers KJ122 and KJ123, creating plasmid pKJ76 carrying ZUO1 without FLAG. Then, the fragment from pKJ76 was replaced by synthesized DNA fragment 3, creating pKJ79. pKJ80 was generated by subcloning the *PstI-BamHI* fragment from pKJ79. pKJ66 was constructed by replacing the *HindIII-FseI* fragment of pGL4-CMV by a fragment amplified by PCR from yeast cDNA with primers KJ110 and KJ111. pKJ67 was constructed by replacing the *EcoRI-FseI* fragment of pGL4-CMV by a fragment amplified by PCR from yeast cDNA with primers KJ112 and KJ113. pKJ68 was constructed by replacing the *BamHI-SalI* fragment of pGEX-5-X-3 with a fragment amplified by PCR from yeast cDNA using primers KJ108 and KJ109.

### Yeast strain creation

All yeast strains (incl. their genotypes) and primers used are listed in Supplemental Tables S3 and S4.

Yeast deletion strains lacking candidate translation factor genes were sourced from the Euroscarf yeast deletion collection. Yeast double deletion strains were constructed using deletion cassettes carrying the *hphNT1* and *natMX6* genes, carried by plasmids pZC3 and pZC4. The deletion cassettes for *SSZ1* deletion were amplified using PCR from their corresponding plasmids, using primers KJ114 and KJ115. To create YKJ12 (*rai1*Δ *ssz1*Δ) and YKJ9 (*ssz1*Δ *zuo1*Δ), the parental yeast strains EUR3 (*rai1*Δ) and EUR78 (*zuo1*Δ) were then transformed with the deletion cassettes targeting *SSZ1* to confer antibiotic resistance and selected using Nourseothricin (Jena Bioscience), or Hygromycin B (Carl Roth GmbH), respectively, on YPD agar medium. Incorporation of the cassettes was verified by colony PCR (Lõoke et al. 2011) using confirmation primers KJ116 and PB238.

### Yeast growth assays

For yeast spot assays, yeast strains were spotted onto minimal synthetic defined (SD) medium plates in five serial 10-fold dilutions (starting with OD_600_ 0.5) and grown for 48 h at 30°C.

For yeast doubling times, yeast strains were grown in liquid culture overnight to stationary phase and diluted to OD_600_ = 0.1 the next morning. Diluted cultures were grown in 24 well plates in 1 mL of minimal SD media supplemented with leucine, methionine and uracil (additionally, also with 50 mM 3-AT) at 30°C with high agitation in the Eon Microplate Spectrophotometer (BioTek). OD_600_ was measured every 15 min. For doubling time calculation, the formula: Doubling Time = [LN(2)/LN(OD_600_/OD_600_)] was used. Each experiment was performed in three biological replicates. Each biological replicate was the average of three technical replicates.

### Yeast treatment and lysis for western blot

For Supplemental Figure S1, yeast were grown to OD ∼0.4–0.6 and 4 absorbance units (AU) of OD_600_ were harvested. The rest of cultures was treated with 50 mM 3-AT for 75 min and 4 AU of OD_600_ were harvested. Yeast were lysed (Kushnirov 2000) with small modifications. In short, harvested yeast cells were pelleted. Pellets were then washed with dH_2_O, resuspended in 100 µL of dH_2_O + 100 µL of 0.2M NaOH and incubated for 5 min at RT. Cells were then pelleted again. Pellets were resuspended in 50 µL of 1× SDS-PAGE Sample Buffer, vortexed and incubated at 98°C for 5 min. Before loading the SDS-PAGE gel, samples were clarified by centrifugation at 18,000*g* for 30 sec. Five microliters of each sample was loaded in one lane.

### Polysome profiling and fraction collection

For polysome profiling, yeast cultures were grown to OD_600_ ∼ 0.8 in YPD media at 30°C 230 rpm. Then, they were treated with cycloheximide (Sigma Aldrich; 50 μg/1 mL of culture) for 10 min and immediately put on ice for 5 min. The pellets were washed with dH_2_O and then resuspended in 1 mL of GA^+^ buffer (20 mM Tris-HCl [pH 7.5], 50 mM KCl, 10 mM MgCl_2_, 1 mM DTT, 5 mM NaF, 50 μg/mL cycloheximide). After centrifugation, the pellet was again resuspended in 300 μL of GF buffer (20 mM Tris-HCl [pH 7.5], 50 mM KCl, 10 mM MgCl_2_, 1 mM DTT, 5 mM NaF, 50 μg/mL cycloheximide, cOmplete EDTA-free Protease Inhibitor mix [Roche]) per 50 mL of cell culture and cells were broken using glass beads. Afterward, the lysate was clarified at 13,500*g*/10 min/4°C. Eight AU OD_260_ were loaded on a 5%–50% sucrose gradient and separated by ultracentrifugation at 39,000 rpm for 2.5 h using the SW41Ti rotor. The gradients were scanned at A254 nm using the Biocomp Gradient Station (Biocomp Instruments).

For fraction collection, yeast strains transformed with FLAG-Ssz1p, FLAG-Rai1p and FLAG-Zuo1p plasmids were grown in minimal SD media supplemented with histidine, methionine and uracil to OD_600_ ∼ 0.9–1.0 at 30°C 230 rpm. Then, cells were put on ice, and 1% formaldehyde (Sigma Aldrich) was added as a crosslinking agent and incubated for 60 min on ice. Afterward, 10 mL of 2.5M Glycine/100 mL of culture was added. The pellets were then resuspended in GF buffer, centrifuged, again resuspended in 300 μL of GF buffer per 50 mL of cell culture, and broken using glass beads. For Figure 5A–C, 20 AU OD_260_ were loaded on a 5%–50% sucrose gradient and separated by ultracentrifugation at 39,000 rpm for 2.5 h using the SW41Ti rotor. For Supplemental Figure S3, a 7%–30% gradient was used. Fractions were collected using the Biocomp Gradient Station (Biocomp Instruments). Each fraction was precipitated with 96% ethanol (2× volume of the fraction) at −20°C overnight, then centrifuged at 21,000*g*/1 h/4°C. The resulting pellets were then resuspended in 100 μL of 1× SDS-PAGE Sample Buffer and 30 μL/well was used for western blot analysis. Thirty microliters of WCE was used as input.

### Western blot

Samples were resolved through SDS-PAGE followed by western blotting. For SDS-PAGE, Criterion TGX Gels were used. After wet transfer, membranes were blocked in 5% milk for 60 min and incubated with primary antibodies at 4°C overnight. The primary antibodies used were anti-FLAG M2 (Sigma Aldrich F1804, dilution 1:500), anti-TIF32 (sourced from Lon Phan, NCBI, USA; dilution 1:1000), anti-PGK1 (Abcam 22C5D8, dilution 1:1000), anti-P-Ser51 eIF2α (Cell Signalling 9721, dilution 1:500), anti-total eIF2α (sourced from Thomas Dever, NIH Bethesda, USA; dilution 1:1000), and anti-Rps26 (sourced from Vikram Panse, University of Zurich, Switzerland; dilution 1:1000). Membranes were washed 3 × 5 min in TBS-T buffer and then incubated in secondary antibodies at room temperature for 90 min. The secondary antibodies used were Amersham ECL Anti-Mouse IgG HRP-linked (Cytiva NA931V, lot# 17853437, dilution 1:2000), Amersham ECL Anti-Rabbit IgG HRP-linked (Cytiva NA934V, lot #18227506, dilution 1:2500) (for Fig. 5 and Supplemental Fig. S3) and BioRad Goat anti-Rabbit IgG HRP-conjugate (1706515, dilution 1:10,000) (Supplemental Fig. S1). Afterward, the membranes were washed 3 × 5 min in TBS-T. For Figure 5 and Supplemental Figure S3, signal was developed using SuperSignal West Femto Maximum Sensitivity Substrate (Thermo Fisher Scientific) and imaged using G-Box (Syngene). For Supplemental Figure S1D, the signal was developed using Amersham ECL Western Blotting Detection Reagent (Cytiva) and imaged on Agfa Medical X-Ray film using the Optimax medical X-Ray film processor (Protec).

### Coimmunoprecipitation

Yeast strain *rai1*Δ*/ssz1*Δ was transformed with *FLAG-RAI1/FLAG-SSZ1* carrying plasmid, then grown along with *rai1*Δ*/ssz1*Δ (no plasmid) in SD media supplemented with histidine, methionine and uracil (and leucine) to OD_600_ ∼ 0.8 at 30°C 230 rpm. The pellets were washed with 25 mL of cold FLAG buffer (20 mM TRIS [pH 7.5], 200 mM KCl, 5 mM MgAc), then weighed and resuspended in cold FLAG buffer with protease inhibitors (Aprotinin 1 μg/mL, Leupeptin 1 μg/mL, Pepstatin 1 μg/mL, 1 mM PMSF, cOmplete EDTA-free Protease Inhibitor mix [Roche], 1 mM DTT). The amount of buffer used was 1 mL/g of pellet weight. The cells were broken using glass beads and the lysate was subsequently cleared 2× at 18,400*g*/10 min/4°C. For the pulldown, ANTI-FLAG M2 Affinity Gel (Millipore) beads slurry was washed 3× with cold FLAG buffer (with protease inhibitors). Afterward, whole cell lysate was added (0.2 mg of total protein adjusted to 1 mL volume with FLAG buffer) to the beads (2.5/10 μL of beads slurry for Rai1/Ssz1, respectively) and incubated for 2 h at 4°C on a rotator. Then, the beads were washed 3× with 1 mL of cold FLAG buffer with 0.2% Triton X-100 (Sigma Aldrich), 1× with cold FLAG buffer without Triton X-100 and 2× with cold 50 mM TRIS (pH 8.0) and finally resuspended in 50 μL of 50 mM TRIS (pH 8.0) to be sent for MS analysis. Total protein concentrations were determined using Bradford assay.

### Protein digestion

Beads were resuspended with 100 µL of 100 mM TEAB containing 2% SDC. Cysteins were reduced with 10 mM final concentration of TCEP and blocked with 40 mM final concentration of chloroacetamide (60°C for 30 min). Samples were cleaved on beads with 1 µg of trypsine at 37°C overnight. After digestion, samples were centrifuged and supernatants were collected and acidified with TFA to 1% final concentration. SDC was removed by extraction to ethylacetate (Masuda et al. 2008). Peptides were desalted using in-house made stage tips packed with C18 disks (Empore) according to Rappsilber et al. (2007).

### nLC-MS 2 analysis

Nano Reversed phase column (IonOpticks Aurora Ultimate XT 25 cm × 75 µm ID, C18 UHPLC column, 1.7 µm particles, 120 Å pore size) was used for LC/MS analysis. Mobile phase buffer A was composed of water and 0.1% formic acid. Mobile phase B was composed of acetonitrile and 0.1% formic acid. Samples were loaded onto the trap column (Acclaim PepMap300, C18, 5 µm, 300 Å wide pore, 300 µm × 5 mm, 5 cartridges) for 4 min at 15 μL/min. Loading buffer was composed of water, 2% acetonitrile and 0.1% trifluoroacetic acid. Peptides were eluted with Mobile Phase B gradient from 4% to 35% B in 60 min. Eluting peptide cations were converted to gas-phase ions by electrospray ionization and analyzed on a Thermo Orbitrap Ascend (Q-OT- qIT, Thermo). Survey scans of peptide precursors from 350 to 1400 m/z were performed at 120K resolution (at 200 m/z) with a 5 × 10^5^ ion count target. Tandem MS was performed by isolation at 1,5 Th with the quadrupole, HCD fragmentation with normalized collision energy of 30, and rapid scan MS analysis in the ion trap. The MS/MS ion count target was set to 10^4^ and the max injection time was 35 msec. Only those precursors with charge state 2–6 were sampled for MS/MS. The dynamic exclusion duration was set to 45 sec with a 10 ppm tolerance around the selected precursor and its isotopes. Monoisotopic precursor selection was turned on. The instrument was run in top speed mode with 2 sec cycles (Hebert et al. 2014).

### Protein expression and purification

The *E. coli* strain BL21 Star (DE3) (Invitrogen) was used for expression and purification of GST-tagged Ssz1 protein. Expression of GST-Ssz1 was induced by 10 mM IPTG (Serva) overnight at 16°C 230 rpm in LB media. Pellet was washed in cold dH_2_O and resuspended in 5 mL of cold PBS (pH 7.5, 137 mM NaCl, 2.7 mM KCl, 10 mM Na_2_HPO_4_, 1.8 mM KH_2_PO_4_). Cells were broken using glass beads, and 1.5% Triton X-100 (Sigma Aldrich) was added. The mixture was incubated on a rotator for 30 min at 4°C and then centrifuged at 21,000*g*/10 min/4°C. Gluthathione Sepharose beads (50% slurry; GE Healthcare) were washed 3× with PBS and 400 μL of the slurry was added into the clarified cell lysate. The lysate and beads were then incubated for 30 min at RT. Afterward, the beads were washed 3× with cold PBS, aliquoted and frozen at −80°C for use in the GST pulldown.

### In vitro GST pulldown

^35^S-labeled proteins were produced using the TnT Quick Coupled Transcription/Translation System (Promega) according to vendor instructions. GST-Ssz1 on Sepharose beads was incubated with ^35^S-Rai1 and ^35^S-Zuo1 for 2 h at 4°C in 300 μL of buffer B (20 mM HEPES [pH 7.5], 75 mM KCl, 0.1 mM EDTA, 2.5 mM MgCl_2_, 0.05% Igepal CA-630, 1 mM DTT, 1% fat-free milk) and then washed 3× with PBS. The proteins were separated by SDS-PAGE, gels were first stained with Simply Blue SafeStain (Invitrogen) and then subjected to autoradiography.

### Statistical analysis

For all reporter assays, all data were tested for normality by the Shapiro–Wilk test. After confirming normality, the parametric unpaired, two-tailed Welch's *t*-test was used; (****) *P* < 0.0001, (***) *P* < 0.001, (**) *P* < 0.01, (*) *P* < 0.05, (ns) nonsignificant. All individual data points are shown as part of bar plots. The SD was calculated from all biological replicates for each construct in each strain. Statistical analysis and visualization were performed in GraphPad Prism, version 9.4.1 (GraphPad Software). All replicates shown in the reporter assays graphs were independent transformants—biological replicates.

All MS data were analyzed and quantified with MaxQuant software (version 2.3.1.0) (Cox et al. 2014). The false discovery rate (FDR) was set to 1% for both proteins and peptides and we specified a minimum peptide length of seven amino acids. The Andromeda search engine was used for the MS/MS spectra search against the *S. cerevisiae* database (downloaded from Uniprot.org in February 2024, containing 6060 entries). Enzyme specificity was set as C-terminal to Arg and Lys, also allowing cleavage at proline bonds and a maximum of two missed cleavages. Carbamidomethylation of cysteine was selected as fixed modification and N-terminal protein acetylation and methionine oxidation as variable modifications. Data analysis was performed using Perseus 1.6.15.0 software (Tyanova et al. 2016).

For volcano plot creation, the VolcaNoseR tool was used (Goedhart and Luijsterburg 2020). Log_2_FC threshold was set as −1 and +1 (corresponding to twofold change) and threshold −Log *P*-value was set as 2 (corresponding to *P* = 0.01).

## DATA DEPOSITION

All data from this study are available within this paper and its Supplemental Material. All plasmids and strains used in this study are available upon request from the authors. Source data for graphs presented in Figures 2, 3, and Supplemental Figure S1 can be found in Supplemental Data 1. Source data for MS in Figure 6 can be found in Supplemental Data 2.

## SUPPLEMENTAL MATERIAL

Supplemental material is available for this article.

## Figures and Tables

**Kristína Jendruchová:**
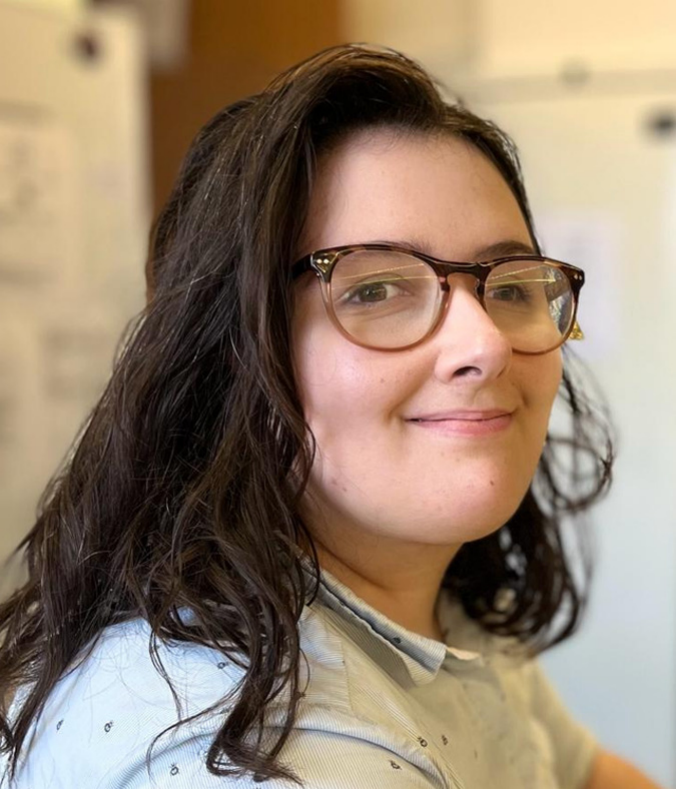


## MEET THE FIRST AUTHOR

***Meet the First Author(s)* is an editorial feature within *RNA*, in which the first author(s) of research-based papers in each issue have the opportunity to introduce themselves and their work to readers of *RNA* and the RNA research community. Kristína Jendruchová is the first author of this paper, “Yeast Rai1p and the RAC complex (Ssz1p/Zuo1p) modulate uORF-mediated *GCN4* translational control under stress conditions.” Kristína is currently working as a postdoc at the Laboratory of Genome Dynamics under Dr. Hana Hanzlikova (Institute of Molecular Genetics, Czech Academy of Sciences). The position and project are a joint endeavor with the Valášek lab. They are investigating regulatory links between the ribosome, its associated factors, and the DNA damage response.**

**What are the major results described in your paper and how do they impact this branch of the field?**

*GCN4* is a transcription factor that orchestrates the yeast stress response, similarly to the mammalian ATF4. Its translation is regulated by four upstream ORFs (or “uORFs”) in the gene's 5′ leader sequence. These uORFs influence translation of the main CDS through translation reinitiation. Upon stress, phosphorylation of the translation initiation factor eIF2α changes the dynamics of translation (re)initiation, which results in a boost in Gcn4 protein production. However, these processes are also regulated by other *trans*-acting factors.

Our screening revealed three new factors that regulate reinitiation at the *GCN4* uORFs—Rai1p, Ssz1p and Zuo1p (the latter two form the ribosome associated complex). Their absence results in improper induction of Gcn4p translation, and a reduction of yeast fitness upon stress, similar in the absence of each of the three factors. Furthermore, we are the first to show Rai1p may have a role in cytoplasmic translation, since it's only been known in translation as a nuclear ribosome biogenesis factor before. It would be interesting to see in the future if the same could apply to the factors’ mammalian counterparts and ATF4.

**What led you to study RNA or this aspect of RNA science?**

In general, I adore how diverse the RNA world is—transcription, translation, splicing, modifications, stability, regulation … and anything in between. With RNA, you're bound to hear about many different topics, and you never know when the pieces may come together to form new connections. It's also quite challenging at the same time. When I was picking my PhD topic, the ribosome seemed like an intriguing little machine to study (and it still is!), and I was always attracted to regulatory elements of gene expression.

**During the course of these experiments, were there any surprising results or particular difficulties that altered your thinking and subsequent focus?**

The project started more as a general screen of factors that could influence translation reinitiation and ribosome recycling—in fact, the same reporter system that was used here, we also used in another project investigating the function of 40S recycling factors. So we were most surprised when we found that deletion of all three factors led to a very similar defect in Gcn4p induction under stress, even when some of them had the opposite phenotypes in reinitiation after solitary uORFs. That made us pivot more into this direction. At the same time, it points to some more intricate regulatory mechanisms at play here. It also stresses the importance of these factors for the proper functioning of the *GCN4* regulatory system as a whole, not just as a sum of its parts.

**What are some of the landmark moments that provoked your interest in science or your development as a scientist?**

I had an interest in science since I was a little kid, but for molecular biology specifically. I think it was learning about Mendel's laws and the central dogma in high school that jump-started my interest and made me apply for university in the genetics program. As a scientist, a lot of my development came from just talking about science with more experienced people. Not only about specific experiments, but about their own personal philosophies, opinions, stances. Then it often comes down to synthesizing the best aspect of each and incorporating it into your own development. I also feel like I got a lot of inspiration from reading older landmark papers and their much more elegant experimental design—a lot can be learned from those principles even today.

**If you were able to give one piece of advice to your younger self, what would that be?**

I have two, one abstract and one practical. One, listen to your intuition, and two, write down everything, because you won't remember. Those two things I had to learn the hard way even if they seem obvious to some people. I think following one's intuition is an underutilized aspect of humanity in the sciences—and following your hunches often turns out to be supported by the data in the end.
